# Enhanced Adult Neurogenesis Increases Brain Stiffness: *In Vivo* Magnetic Resonance Elastography in a Mouse Model of Dopamine Depletion

**DOI:** 10.1371/journal.pone.0092582

**Published:** 2014-03-25

**Authors:** Charlotte Klein, Elisabeth G. Hain, Juergen Braun, Kerstin Riek, Susanne Mueller, Barbara Steiner, Ingolf Sack

**Affiliations:** 1 Department of Neurology, Charité - University Medicine Berlin, Berlin, Germany; 2 Institute of Medical Informatics, Charité - University Medicine Berlin, Berlin, Germany; 3 Department of Radiology, Charité - University Medicine Berlin, Berlin, Germany; 4 Center for Stroke Research Berlin, Berlin, Germany; Charité University Medicine Berlin, Germany

## Abstract

The mechanical network of the brain is a major contributor to neural health and has been recognized by in vivo magnetic resonance elastography (MRE) to be highly responsive to diseases. However, until now only brain softening was observed and no mechanism was known that reverses the common decrement of neural elasticity during aging or disease. We used MRE in the 1-methyl-4-phenyl-1,2,3,6-tetrahydropyridine hydrochloride (MPTP) mouse model for dopaminergic neurodegeneration as observed in Parkinson’s disease (PD) to study the mechanical response of the brain on adult hippocampal neurogenesis as a robust correlate of neuronal plasticity in healthy and injured brain. We observed a steep transient rise in elasticity within the hippocampal region of up to over 50% six days after MPTP treatment correlating with increased neuronal density in the dentate gyrus, which could not be detected in healthy controls. Our results provide the first indication that new neurons reactively generated following neurodegeneration substantially contribute to the mechanical scaffold of the brain. Diagnostic neuroimaging may thus target on regions of the brain displaying symptomatically elevated elasticity values for the detection of neuronal plasticity following neurodegeneration.

## Introduction

Magnetic resonance elastography (MRE) has been developed over the last few years as a non-invasive tool to evaluate the elasticity of biological tissues [1]. The presence of the skull has always prevented manual palpation of the brain, but MRE now offers the possibility to assess brain consistency under physiological and pathological conditions by in-vivo imaging [2]–[7]. In the brain, the viscoelastic properties are determined by neurons, glial cells [8] and extracellular matrix in addition to fluid flow of interstitial fluid, CSF and blood [9]. Disruption of this complex system by pathological processes provokes mechanical responses, which are influential to the progression of the disease but also of potential value for its diagnosis and clinical assessment. However, the biophysical mechanisms behind an alteration of the mechanical properties of tissue are entirely unknown in the brain.

It has just recently been discovered that brain elasticity is reduced in the course of physiological aging [10] and in diseases such as normal pressure hydrocephalus (NPH) [11], Alzheimer’s disease (AD) [12] and multiple sclerosis (MS) [13], [14]. First steps to correlate these findings with the histopathology have been taken quite recently by Schregel and colleagues inducing reversible toxic demyelination in the mouse [15] and by Riek and co-workers who studied the effect of inflammation in a mouse model of experimental autoimmune encephalitis (EAE) [16]. Both groups observed a marked decrease of viscoelastic constants similar to what has been detected in patients with NPH, AD and MS. Of particular interest is a very recent study by Freimann and co-workers demonstrating a clear correlation of brain tissue softening with reduced neuronal density after middle cerebral artery occlusion (MCAO) in mice, which is a commonly used stroke model [17]. It is remarkable that all pathophysiological processes studied by cerebral MRE so far exhibited a rather unspecific reduction in either elasticity or viscosity or both. Inversely, no neural alteration has been observed associated with an increase of viscoelastic constants. Potentially, such a disease-related process would appear highly significant in diagnostic MRE since it would be distinguishable from the general pattern of tissue softening reported in the literature. Based on previous work that showed the correlation between neuronal density and macroscopic brain stiffness by inducing neuronal loss [17], we hypothesize that the generation of new neurons would increase the macroscopic elasticity of the brain. Given that this hypothesis is corroborated, our study would provide an indication about the close relationship between brain mechanical constants and neuronal network density.

The generation of new neurons should be apparent in regions with high cellular turnover. In the adult brain, new nerve cells are generated in the subgranular zone (SGZ) of the hippocampal dentate gyrus (DG) at substantial levels throughout lifetime. Here, neural precursor cells characterized by the expression of the intermediate filament nestin [18] continuously proliferate and mature into functionally integrated cells in the granular cell layer (GCL) via a multistep process termed adult neurogenesis [19]–[21]. It has been shown that a homeostasis of neurotransmitters such as dopamine plays a key role in the regulation of adult neurogenesis and the maintenance of a so-called neurogenic niche. Alterations in dopamine levels as observed in Parkinson’s disease (PD) and its animal models result in significant quantitative changes of newly generated neural precursor cells and mature neurons in the DG [22]–[30].

Therefore, we applied MRE to such a mouse model of dopamine depletion, which is induced by the neurotoxin 1-methyl-4-phenyl-1,2,3,6-tetrahydropyridine hydrochloride (MPTP) leading to degeneration of dopaminergic neurons in the substantia nigra pars compacta (SNpc) with a subsequent dopamine deficit in the striatum, hippocampus and other brain areas that are innervated by dopaminergic fibers from the SNpc [31]–[35]. Cellular changes in the DG of the hippocampus as a consequence of the induced dopamine deficit were visualized by labelling newly generated cells in the SGZ with specific mitotic and neuronal markers, and characterized and quantified using confocal microscopy. To also determine alterations in the total cell number, cells of the GCL in the DG including the SGZ were counted using stereological microscopy. Hence, this animal model of dopamine depletion provides the possibility to examine how alterations of the neuronal matrix in certain brain areas resulting from a dopamine deficit relate to MRE measurements, and to expand the utility of MRE as a tool to monitor cellular changes in the pathology of neurodegenerative diseases.

## Materials and Methods

### Animals

A total of 60 eight to ten weeks old female transgenic C57Bl/6 mice (Forschungseinrichtung für experimentelle Medizin, FEM, Berlin, Germany), expressing the green fluorescent protein (GFP) under the nestin promoter, were group-housed (n = 5) in a temperature- and humidity-controlled colony room and maintained on a light/dark cycle of 12/12 h (lights on at 6 am) with ad libitum access to rodent lab chow and water. All experiments were approved by the local animal ethics committee (Landesamt für Gesundheit und Soziales, Berlin) and were carried out in accordance with the European Communities Council Directive of 24 November 1986 (86/609/EEC).

### Group Design and Experimental Procedure

The mice were randomly assigned to four groups in total - three for histology and one for MRE. The MRE group (n = 5) was investigated first without any modifications for obtaining reference (baseline) data; then MRE was applied at five time-points after NaCl treatment at 3, 6, 10, 14 and 18 days post injection (dpi), followed by MPTP treatment and MRE measurements again at 3, 6, 10, 14 and 18 dpi. The last MRE measurement of NaCl-treated mice (18 dpi) also served as baseline data of MPTP-treated mice.

**Figure 1:**
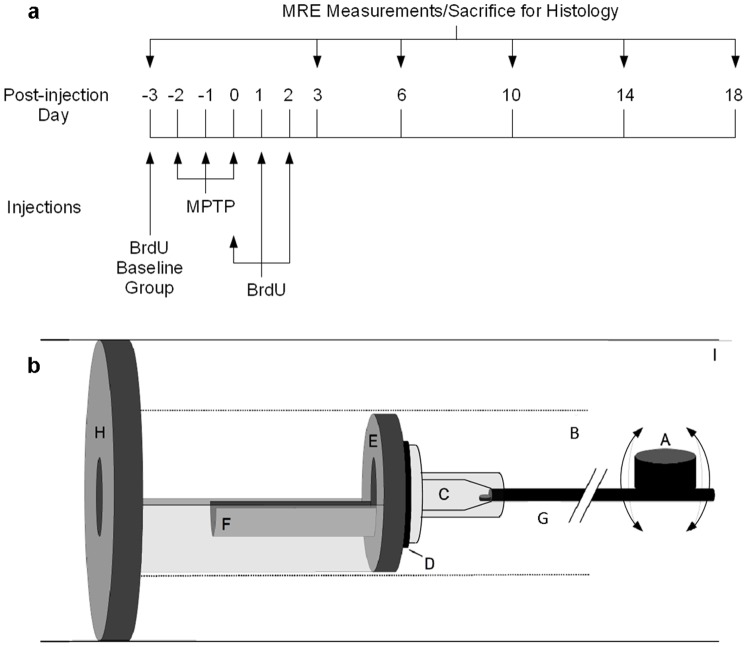
Timeline of the study design and schematic of the mouse MRE apparatus. The timeline (a) of MPTP and BrdU injections, time-points of MRE measurement and sacrifice for histology. A schematic (b) of the mouse MRE apparatus: (A) driving coil, (B) magnet bore, (C) respiratory mask, (D) rubber bearing, (E) retaining bracket, (F) mouse bed, (G) carbon fiber piston, (H) plastic disk, and (I) Lorentz coil (modified from [16]).

The histology groups (not investigated by MRE) comprised untreated mice (baseline; n = 5) and treated animals either with MPTP (MPTP; n = 25) or with NaCl (Control, CTR; n = 25). The treated groups (MPTP and CTR) were further subdivided into groups of five, which were sacrificed at 3, 6, 10, 14 and 18 days after the MPTP/NaCl treatment period. Figure 1a summarizes the timeline of the injections, MRE experiments and histology.

### MPTP Model

1-methyl-4-phenyl-1,2,3,6-tetrahydropyridine hydrochloride (MPTP, Sigma-Aldrich, Steinheim, Germany) was dissolved in 0.9% NaCl.

For lesioning, a protocol previously applied was followed [27], [28]. Briefly, mice received three single intraperitoneal (i.p.) injections of MPTP (20 mg/kg body weight; in total 60 mg/kg) on three consecutive days every 24 hours. Control animals were treated with injections of 0.9% NaCl instead for the same time period. Control mice of the baseline group remained untreated.

### BrdU Injections

Bromodesoxyuridine (BrdU, Sigma-Aldrich, Steinheim, Germany), used here as the mitotic marker to label proliferating cells, was dissolved in 0.9% NaCl and filtered. Animals received three single i.p. injections of BrdU (50 mg/kg body weight; in total 150 mg/kg) on three consecutive days every 24 h. BrdU treatment started at the final day of MPTP-injections.

### Magnetic Resonance Elastography (MRE)

#### Mechanical stimulation

Mouse brains were mechanically stimulated as previously illustrated [16]. A schematic of the experimental setup is shown in Figure 1b. Briefly, the vibration source was an electromagnetic coil, attached to a carbon fiber piston, the end of which was mounted to the respiratory mask with a bite-bar transducer. The transducer was gimballed through a rubber bearing and retaining bracket at the temperature-controlled mouse bed. The entire setup was held in the centre of the magnet bore by a plastic disk. Vibrations were produced by applying a sinusoidal current of 900 Hz frequency to an air-cooled Lorentz coil in the fringe field of the MRI scanner. Frequency amplitude and number of sinusoidal oscillation cycles were controlled by an arbitrary function generator connected via an audio amplifier to the driving coil. The main polarization of the vibration was transverse to the principal axis of the magnet field, with amplitudes in the order of tens of micrometers.

#### Data acquisition and analysis

As previously described [16], [17], all measurements were performed on a 7 tesla scanner (Bruker PharmaScan 70/16, Ettlingen, Germany) running ParaVision 4.0 software and using a 20 mm diameter 1H-RF-quadratur mouse head volumecoil. The vibration was initiated by a trigger pulse from the control unit of the scanner, the timing of which was defined by a customized FLASH sequence. The imaging sequence was modified for MRE by sinusoidal motion sensitizing gradient (MSG) in the through-plane direction, as described elsewhere [16]. The MSG strength was 285 mT/m with a frequency of 900 Hz and 9 periods. To compensate for static phase contributions, phase difference images were calculated from two images differing in the sign of the MSG. Further imaging parameters were: a 128×128 matrix, 25 mm FoV, 14.3 ms echo time (TE), 116.2 ms repetition time (TR), eight dynamic scans over a vibration period, one transverse 2-mm slice, and an acquisition time of 20 min.

**Figure 2:**
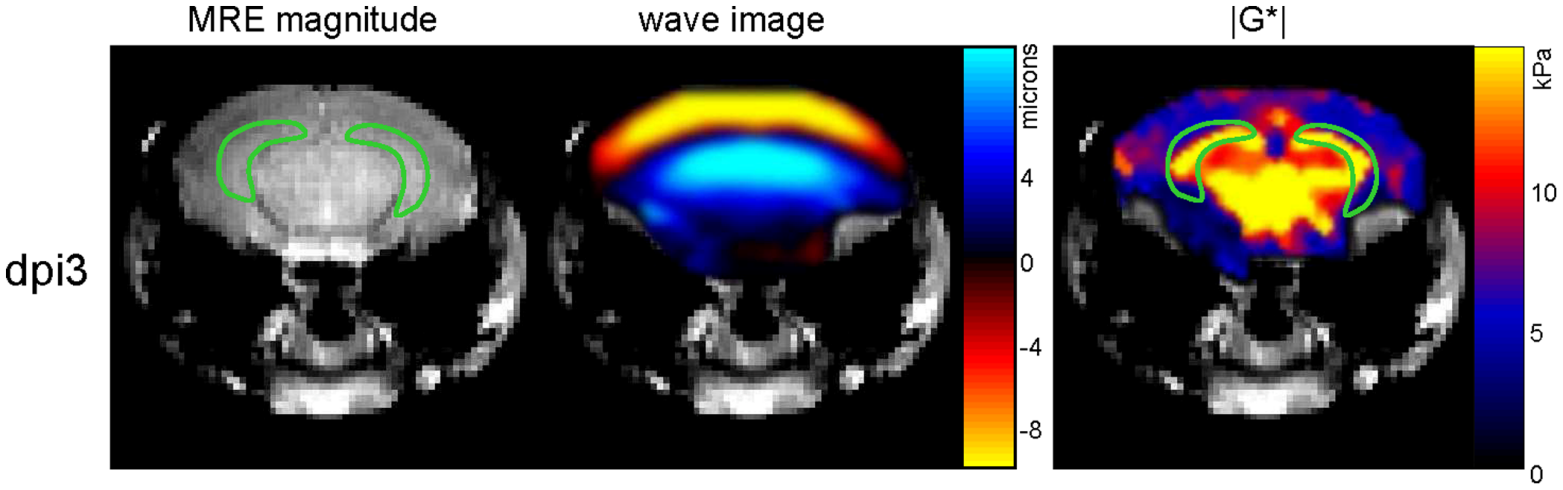
Representative images of the magnitude MRE signal, shear waves and the magnitude complex modulus (|G*|) in a mouse. The green line demarcates the chosen region of interest in the hippocampus.

Complex wave images (Figure 2) corresponding to the harmonic drive frequency were calculated by temporal Fourier transformation of the unfolded phase-difference images and filtered for suppressing noise and compression wave components [16], [36]. The pre-processed 2D scalar wave fields were analyzed for the complex shear modulus G* by algebraic Helmholtz inversion [37]. Then, G* was spatially averaged over two regions of interest (ROI’s), i) the whole brain parenchyma displayed in the image and ii) the hippocampal area (Figure 2), manually segmented by delineating its anatomical structure from MRE magnitude images. The tabulated spatially averaged G*-values are represented by the real part of the complex shear modulus G*, G′ = Re(G*), known as storage modulus, the imaginary part G″ = Im(G*), which is the loss modulus, the magnitude |G*| = abs(G*) and the loss tangent given by φ = arctan(G″/G′). The storage, loss and magnitude moduli are expressed in kilopascals (kPa) while φ is given in radians. In general, G′ relates to the elastic properties of a material, while G″ is a measure of viscosity, which is determined by the density and geometry of the mechanical network in biological tissues. In materials with dominating elastic behaviour, the parameters |G*| and φ represent similar properties as G′ and G″. However, in highly crosslinked biological tissues, the phase angle φ better represents geometrical changes in the mechanical network than G″ [38].

### Perfusion and Tissue Processing

Mice of the histology groups were sacrificed according to the corresponding MRE measurement time-points as depicted in Figure 1a.

They were deeply anesthetized with an overdose of ketamine (ketamine hydrochloride, 100 mg/ml, WDT) and sacrificed by transcardial perfusion with 1 M phosphate buffered saline (PBS) and 4% paraformaldehyde (PFA). Brains were removed and post-fixed overnight in PFA at 4°C and then transferred into 30% sucrose for dehydration for 48 h. Brains were frozen in 2-methyl butane cooled with liquid nitrogen, cut into 40 μm thick coronal sections (Bregma −0.1 mm to −4.04 mm) using a cryostat (Leica CM 1850 UV) and stored in cryoprotectant-containing 24-well plates at 4°C until histological analysis.

### Immunohistochemistry

Immunohistochemistry for the Iba1 antigen as a marker for microglia and macrophages was performed following a well-established staining protocol [28]. Briefly, free-floating 1-in-12 section series were treated with 0.6% H_2_O_2_ to deactivate endogenous tissue peroxidases. After 30 min background blocking with PBS enriched with 3% donkey serum (PBS+), sections were incubated with primary anti-Iba1 (rabbit, 1∶1000, Wako) antibody overnight at 4°C. The next day, after washing with PBS and blocking with PBS+, sections were incubated with biotinylated secondary antibody (anti-rabbit, 1∶250, dianova) for 2 h at room temperature (RT). ABC reagent (Vectastatin ABC Elite Kit, Vector Laboratories) was applied for 1 h. Finally, sections were incubated with Diaminobenzidine (DAB)/peroxidase (Sigma, Germany) in a solution containing 0.3% H_2_O_2_ and 0.01% nickel chloride for at least 5 min at RT. Sections were mounted on microscope slides and coverslipped for later quantification.

### Immunofluorescence

For triple-labelling, free-floating 1-in-12 section series were rinsed and incubated as described above except for H_2_O_2_ pre-treatment but 1 h PBS+ blocking instead. Anti-BrdU (rat, 1∶500, AbD Serotex) anti-GFP (chicken, 1∶250, Novus Biologicals) and anti-NeuN (mouse, 1∶100, Millipore) were used as primary antibodies. BrdU denotes newly generated cells, while BrdU/Nestin (Nestin/GFP) and BrdU/NeuN denote new neural precursor cells and new neurons, respectively. After incubation for 48 h at 4°C, RhodamineX (anti-rat, 1∶250, dianova), Alexa 488 (anti-chicken, 1∶1000, Invitrogen) and Alexa 647 (anti-mouse, 1∶300, dianova.) as secondary antibodies diluted in PBS+ were applied for 4 h at RT. Sections were mounted on microscope slides and coverslipped for later quantification.

For stereological counting of cells in the GCL of the DG including the cells in the SGZ in order to determine its total cell number, separate 1-in-12 series of sections were incubated with the fluorochrome 4′,6-diamidino-2-phenylindole (DAPI), which binds to the DNA thereby labeling cell nuclei in general. For this purpose, sections were incubated with PBS-diluted DAPI (1∶1000, Thermo Scientific) for 7 min and afterwards mounted on microscope slides and coverslipped for later quantification.

### Quantification

Iba1-positive cells of DAB/peroxidase-stained 1-in-12 section series (480 μm apart) from all animals were counted throughout the rostrocaudal extent of the GCL/SGZ, molecular layer (ML) and hilus in the hippocampal formation in both hemispheres using the 40×objective of a light microscope (Axioskop, Zeiss, Germany). In total, four brain slices per animal containing the hippocampus were analyzed. Resulting absolute cell numbers were then multiplied by twelve to obtain the estimated total number of Iba1-positive cells per brain.

BrdU-positive cells in the fluorescent-stained sections were counted as described for Iba1 but in the GCL/SGZ only using a fluorescence microscope (Axioskop, Zeiss, Germany).

Double-labeled cells were quantified by analyzing 50 BrdU-positive cells spread throughout the rostrocaudal extent of the GCL/SGZ of four brain slices per animal for co-expression of BrdU and additional markers (Nestin/GFP and NeuN) using a Leica TCS SP2 confocal microscope (400x and 630x amplification). All images were taken in a sequential scanning mode (z-stacks) to identify superposed cell bodies or nuclei, which appeared artificially merged. Then, the percentages of Nestin/GFP- and NeuN-positive cells in all 50 BrdU-cells were determined. These rations along with the total numbers of BrdU-positive cells were then used to calculate the absolute numbers of doubly labeled cells.

For quantification of absolute numbers of DAPI-stained cell nuclei in the GCL, a Leica DMRE microscope and StereoInvestigator (MicroBrightfield) software were used. The bounderies of the GCL, the region of interest, of five sections per animal were traced at 200×magnification and the thickness of the slices (40 μm) was entered to the software program. The software then randomly arranged counting frames (30 μm×30 μm×30 μm) in a sampling grid (120 μm×100 μm), which was placed over the region of interest. The DAPI-stained cell nuclei were counted at 400×amplification in oil in the counting frames and in an Optical Disector height of 20 μm, which started 5 μm below the top surface. The total number of cells in the GCL was automatically calculated based on the counted cell number, slice interval, counting frame size, sampling grid size, slice thickness and Optical Disector height.

### Statistical Analysis

To test for normal distribution and homogeneous variances, the Kolmogorov-Smirnov test and the Levené test, respectively, were applied. Since group sizes in this study were completely equal (n = 5), for analysis, two-way ANOVAs of the histological data and repeated measures (RM) two-way ANOVAs of the MRE data were conducted, although not all data sets met the assumptions for an ANOVA. In the two-way ANOVA, Treatment represented the between-subjects factor and Time the within-subjects factor. In the RM two-way ANOVA, Time and Treatment by Time represented the within-subjects factors and Treatment the between-subjects factor. Pairwise comparisons applying the Bonferroni test were used to directly compare the two treatment groups within each time-point or the different time-points within each treatment group, respectively. All statistical analysis was conducted using SPSS Statistics 19 for Windows with the level of significance set at 0.05. The diagrams were prepared using GraphPad Prism 5.

## Results

### MPTP-induced Transient Increase of Brain Elasticity and Viscosity

**Figure 3:**
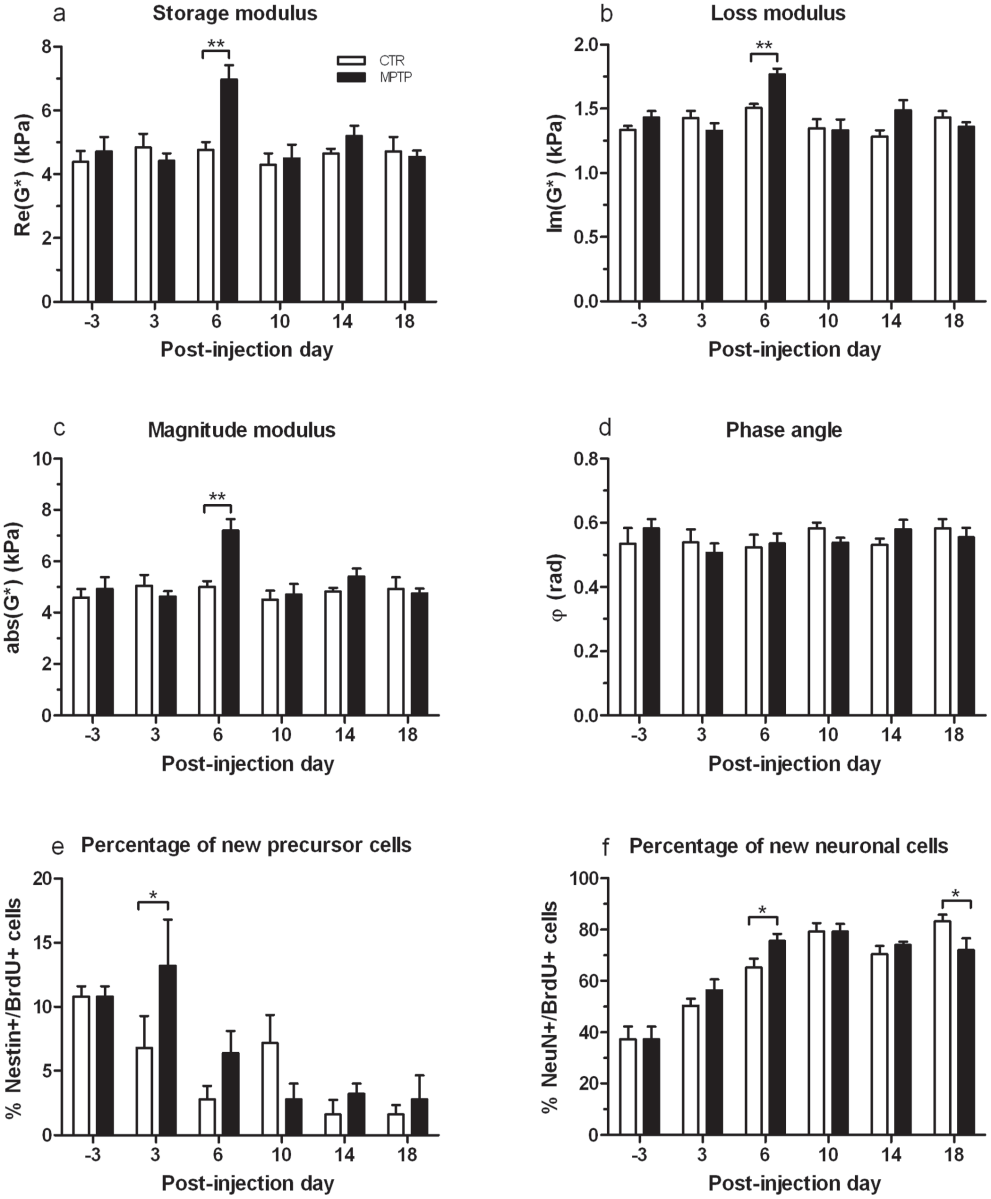
Variation of MRE parameters due to MPTP in the hippocampus of mice and results of cell counts in the DG. MPTP induced a transient increase of brain elasticity and viscosity (a, b and c) at 6 dpi, while the phase angle φ (d) remained unchanged (mean±SEM). Histologically, a transient MPTP-induced increase of new precursor cells (BrdU+/Nestin/GFP+) at 3 dpi (e) and of new neurons (BrdU+/NeuN+) at 6 dpi (f) as percentage of all newborn cells (BrdU+) was found (mean±SEM). *p<0.05, **p<0.01.

We investigated how brain viscoelasticity is affected by a MPTP-induced dopamine deficit. MRE was performed one day before MPTP-treatment (to establish a baseline), and 3, 6, 10, 14 and 18 days after treatment cessation to determine viscoelastic changes as a consequence of dopamine depletion. The results of MRE measurements focussing on the hippocampus, the region that contains a highly neurogenic area (SGZ) modulated by dopamine, are shown in Figure 3 (a–d). No influence of time was seen in the control group for any of the MRE parameters. Mean values (± standard error of the mean, SEM) in the hippocampal region of controls were 4.608 (±0.719) kPa, 1.388 (±0.125) kPa, 4.816 (±0.705) kPa and 0.549 (±0.073) for G′, G″ abs(G*) and φ, respectively. Similar values were found in the entire brain of the control group with 5.234 (±0.564) kPa, 1.447 (±0.87) kPa, 5.432 (±0.553) kPa and 0.574 (±0.034) for G′, G″ abs(G*) and φ, respectively. RM two-way ANOVA showed an overall effect of time on G′ (F(5,40) = 5.239, p<0.001), G″ (F(5,40) = 9.669, p<0.001) and abs(G*) (F(5,40) = 5.689, p<0.001), a treatment by time effect on G′ (F(5,40) = 3.841, p<0.01), G″ (F(5,40) = 4.240, p<0.01) and abs(G*) (F(5,40) = 4.045, p<0.01) in MPTP-treated mice, but no effect of treatment alone on G′ (F(1,8) = 3.758, p = 0.089), G″ (F(1,8) = 1.378, p = 0.274) and abs(G*) (F(1,8) = 3.979, p = 0.081). Pairwise comparison between groups at different days using the Bonferroni test revealed a marked temporary MPTP-induced increase at 6 dpi of G′ (Figure 3a, p<0.01), G″ (Figure 3b, p<0.01) and abs (G*) (Figure 3c, p<0.01) towards 6971 (1019) kPa, 1767 (103) kPa, 7192 (1011) kPa in the hippocampus, which was still significant within the whole-brain region (Figure S1). Relative to baseline values, the changes of G′, G″ and abs(G*) at 6 dpi in the hippocampus were 51%, 27%, and 49% and 29%, 16%, and 28% in the whole brain, respectively. Since the phase angle φ (Figure 3d) remains unchanged by treatment (F(1,8) = 0.363, p = 0.563) during the course of measurements (F(5,40) = 0.573, p = 0.720), the findings suggest that the viscoelasticity of hippocampal tissue is selectively altered transiently after MPTP treatment without influencing the architecture of the cellular matrix.

### MPTP-induced Transient Increase of New Neural Precursor Cells and Neurons

Figure 3 (e and f) shows cell numbers of new neurons (BrdU+/NeuN+) and precursor cells (BrdU/+Nestin/GFP+) relative to the total number of BrdU-positive cells in the SGZ and GCL. Analysis by two-way ANOVA revealed a noticeable time effect on both cell types (BrdU+/Nestin/GFP+: F(5,48) = 9.070, p<0.0001; BrdU+/NeuN+: F(5,48) = 41.910, p<0.0001), but only a marginal interaction of both factors (BrdU+/Nestin/GFP+: F(5,48) = 2,146, p = 0.076; BrdU+/NeuN+: F(5,48) = 2.095, p = 0.082). Pairwise comparisons showed that mice treated with MPTP displayed a larger proportion of new precursor cells at 3 dpi (Figure 3e; p<0.05) and of new neurons at 6 dpi (Figure 3f; p<0.05) than controls regarding the total amount of newly generated cells. However, at 18 dpi, referring to the last day of MRE measurement, MPTP treatment appeared to provoke a reduced neurogenesis expressed as a smaller proportion of new neurons compared to controls (Figure 3f; p<0.05).

**Figure 4:**
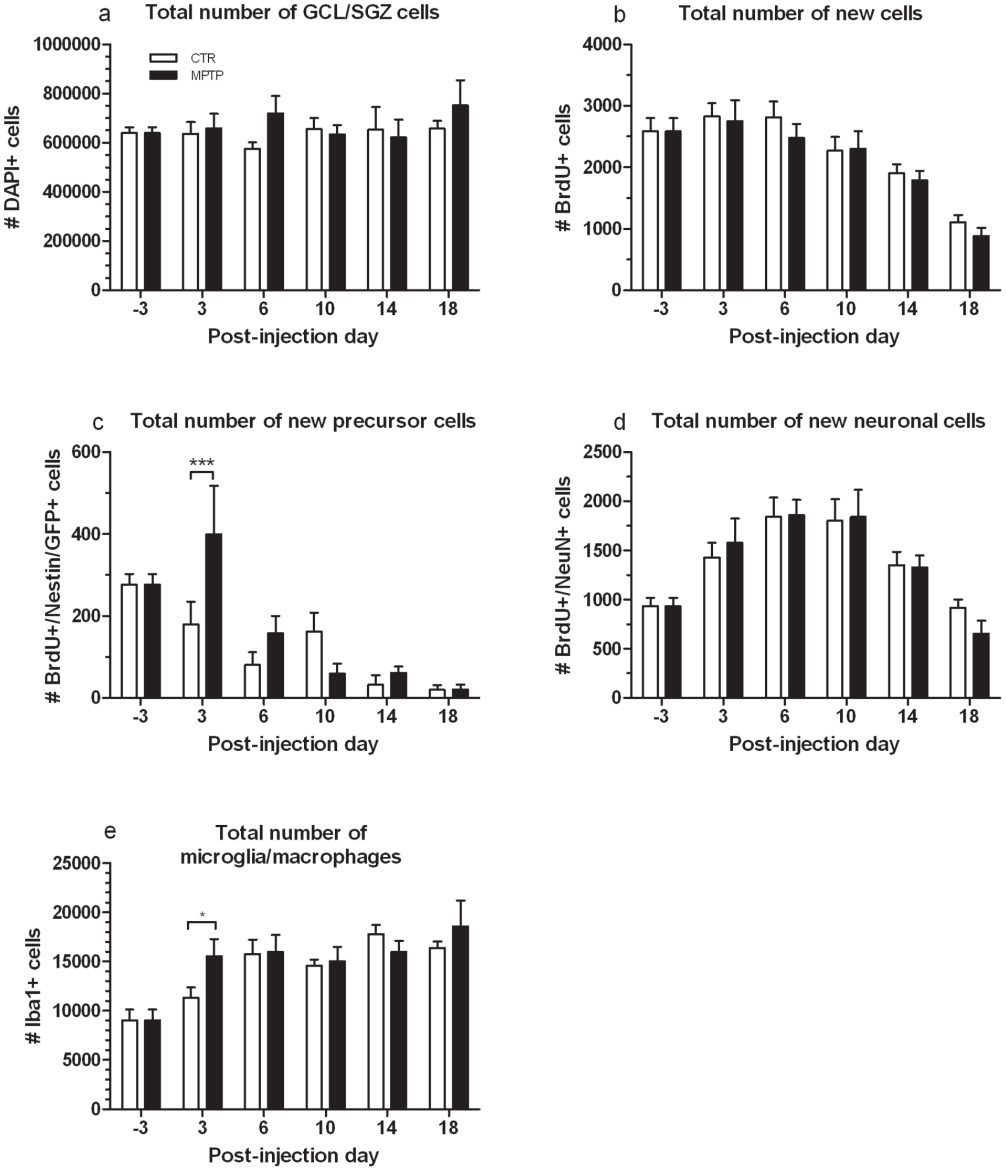
Results of cell counts in the DG. Fluorescence- and DAB-stained brain sections of MPTP-treated and CTR mice at the different time-points of MRE assessment, showing the total number of a) GCL/SGZ cells (DAPI), b) newborn cells (BrdU+), c) new precursor cells (BrdU+/Nestin/GFP+), d) new neuronal cells (BrdU+/NeuN+), and f) microglia and macrophages (Iba1+) in the GCL/SGZ, ML and hilus (mean±SEM). *p<0.05, **p<0.01.

**Figure 5:**
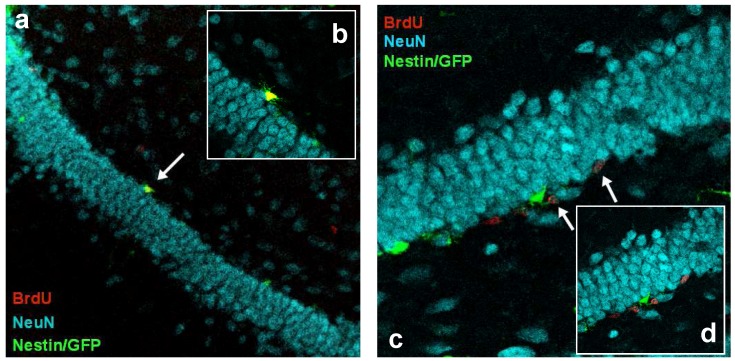
Representative confocal images of doublelabeled BrdU+/Nestin/GFP+ and BrdU+/NeuN+ cells. Mouse brain sections (40 μm) fluorescently stained with BrdU (red), NeuN (blue) and Nestin/GFP (green), showing the granular cell layer of the dentate gyrus with a Nestin/GFP-expressing precursor cell that is also positive for BrdU (a, 200×magnification and b, 630×magnification; arrow) and BrdU-positive cells coexpressing the neuronal marker NeuN (c, 400×magnification and d, 630×magnification; arrows).

Absolute numbers of newly generated cells (BrdU+), new neural precursor cells and new neurons are shown in Figure 4 (b–d). Representative confocal images of BrdU and its colocalization with Nestin/GFP or NeuN, respectively, are shown in Figure 5. A two-way ANOVA revealed a strong influence of time on all three cell types (BrdU: F(5,48) = 19.336, p<0.0001; BrdU/Nestin: F(5,48) = 12.542, p<0.0001; BrdU/NeuN: F(5,48) = 13.920, p<0.0001). Furthermore, the ANOVA also showed a treatment by time effect on newly generated precursor cells (F(5,48) = 2.768, p<0.05). Pairwise comparison showed that in MPTP-treated mice, compared to controls, an increased proliferation of Nestin/GFP cells occurred until 3 dpi (Figure 4c; p<0.001) with a subsequent drop on 6 dpi (p<0.01, compared to MPTP at 3 dpi; not indicated in the figure) that may suggest a transient reactive proliferation of Nestin/GFP precursor cells in response to the neurotoxin as shown before in previous work from our group (27, 28). The observed course of BrdU-, new Nestin/GFP- und new NeuN-positive cell numbers over time generally parallels the suggested different stages during neuronal development including proliferation, survival and maturation [20].

### MPTP-induced Increase of Microglia in the DG Region does not Change the Total Number of Cells in the GCL

The obtained numbers from stereological counting of DAPI-stained GCL/SGZ cells are shown in Figure 4a. Neither an overall effect of treatment (F(1,48) = 1.052, p = 0.31) or time (F(5,48) = 0.382, p = 0.859) alone nor an interaction between these factors (F(5,48) = 0.713, p = 0.616) was found, indicating that the total number of GCL and SGZ cells was not affected by MPTP treatment and there was also no change in the total cell number over time.

Absolute numbers of all Iba1-positive cells in the GCL/SGZ, molecular layer and hilus are shown in Figure 4e. As a two-way ANOVA revealed, only time had an influence on the number of Iba1-positive cells (F(5,48) = 9.635, p<0.0001). Pairwise comparisons showed that treatment groups differed at 3 dpi (p<0.05) with MPTP-treated mice displaying more microglia and macrophages in the DG region than their unaffected controls. However, numbers of Iba1-positive cells in mice treated with MPTP stay high, while controls then reach the same level of microglia and macrophages at 6 dpi.

## Discussion

MPTP-induced degeneration of dopaminergic neurons affects neurogenesis in the hippocampus [24], [28], [29] and leads to changes in neural cell proliferation patterns in wide regions of the brain [24], [25], [27], [39]. We observed for the first time that newly generated neurons in the DG potentially integrate with the mechanical scaffold of brain tissue yielding an apparent invigoration of the viscoelastic lattice with 50% increased shear modulus (G′) in MPTP-lesioned mice at 6 dpi. In comparison, the neuronal density in the SGZ/GCL changed only by 10% (see Figure 3f) thereby highlighting the sensitivity of macroscopic shear modulus to the number and type of cells engaged in the mechanical tissue matrix. This marked increase of shear elasticity as a result of reactively generated neurons induced by a dopamine deficit is in alignment with observations of [17] who demonstrated that, in contrast to our study, brain stiffness in a murine stroke model is reduced due to neuronal loss. Taken together both studies shed light onto the important role of neurons as a supporting structure of the brain’s mechanical scaffold and complements recent results of in vivo MRE in volunteers and patients: A loss of neuronal support in the viscoelastic lattice of the brain may contribute to the disseminated decrease of G′ and G″ observed in the aging brain and in patients with NPH, AD and MS [10]–[14]. Given this pivotal role of neurons for the macroscopic mechanical properties of the brain, it is not surprising that cerebral MRE has been more sensitive to physiological aging than any other MRI method [13], [40].

In general, the sensitivity of in vivo MRE arises from the scaling properties of mechanical constants. The shear modulus of a hierarchical system is determined by crosslinks in each existing level within the tissue’s architecture towards microscopic interactions of cells [38]. Understanding the macroscopic mechanical response of our mouse model requires both knowledge about single-cell properties and how these properties would integrate into the multi-hierarchic lattice of the brain. Single neurons and glia cells taken from mouse brain tissue investigated by scanning force microscopy showed – similar to the bulk tissue – a higher storage modulus than loss modulus (G′>G″) with neurons being generally stiffer than glia cells [41]. This supports our proposed mechanistic explanation that the neuronal network establishes the primary mechanical backbone of the brain. In the bulk tissue, the ratio between elastic and viscose properties (as quantified by our loss tangent φ = arctan(G″/G′)) can give some insight into the alteration of the geometrical arrangement of the mechanical networks. The nonsignificant alteration of φ in our data suggests that newly born neurons do not assemble a new network with own geometry at 6 dpi, shortly after their generation. They rather appear isolated yet as differentiation and maturation of newly generated cells into neurons and their functional integration into the present neuronal network by fully developing axons, dendrites and synaptic links and thus being involved in the structure of the matured viscoelastic lattice takes about four weeks [20], [42]–[45].

There is growing evidence that this process of adult neurogenesis is intimately connected with an intact neurotransmitter homeostasis, since dopamine depletion has been shown to cause disturbances in precursor cell proliferation [24], [27]–[29]. Here, the analyses of newly generated cells in the DG, characterized as cells that incorporated the exogenous mitotic marker BrdU and its co-localization with Nestin/GFP to identify neural precursor cells, showed a marked transient increase of proliferated Nestin/GFP cells at 3 dpi in MPTP-lesioned mice. It is noteworthy that this rise occurred already at 3 dpi, while the transient gain in viscoelasticity was detected three days later at 6 dpi, which makes the proliferated neural precursor cells an unlikely candidate to account for the augmented stiffness. The observed increased number of proliferated precursor cells is in line with previous findings from our laboratory [27], [28] demonstrating an acute and transient rise in new Nestin/GFP-positive cells in the DG and SNpc, respectively, shortly after MPTP treatment. We have previously shown a comparable stimulus-dependent selective regulation of different neural maturation stages in the DG [43], [45]. The observed phenomenon possibly reflects a reactive neural precursor cell proliferation that may be, according to studies on other neurodegenerative processes, interpreted as an endogenous regenerative mechanism of the hippocampus to counteract neuronal injury in terms of keeping the endogenous stem-like cell pool at a stable level [46]–[49]. In the present study, the proportion of new neurons among all BrdU-positive cells at 6 dpi was higher in MPTP-lesioned mice than in unaffected animals. This finding suggests that the previous reactively generated precursor cells maturate into neurons, which further supports the intended regenerative potential of the hippocampus. Unlike the Nestin/GFP-positive precursor cells, the increased neuron density three days later at 6 dpi, arisen from the enhanced precursor cell population in the DG, might have provoked the higher viscoelasticty values of the brain tissue at the same time-point. After this cellular gain up to 6 dpi, the proportion of new neurons slightly decreased until 18 dpi. From this, one could hypothesize that the pathological effect of MPTP is partially compensated by a transiently boosted proliferation of precursor cells without an increased net neurogenesis [29], [39]. Additionally, although MPTP treatment modulated precursor cell proliferation and neurogenesis, it had no effect on the total amount of newborn BrdU-positive cells in the DG, which is in line with findings from other groups [29], [39].

The neurotoxin MPTP is not only known to cause disturbances in neural cell proliferation and differentiation due to dopamine depletion but to also evoke an inflammatory reaction in affected brain areas including accumulation of microglia, lymphocytic infiltration and an increase in cytokine production [50], which might have been also a cause for the observed transient increase in brain elasticity. We addressed this issue by evaluating the number of microglia and macrophages in the DG area, which comprised the GCL/SGZ, molecular layer and hilus. We detected a peak in microglia and macrophages in MPTP-treated at 3 dpi, where no change in MRE measurements was observed. Although it appears unlikely that these inflammation-associated cell types contributed to the noticeable increase in brain viscoelasticity of MPTP-lesioned mice at 6 dpi, we can not preclude an inflammatory rise in extracellular fluid inducing an edematous swelling at a later time-point to be accountable for the observed brain stiffening. Due to the applied fixation method of the brain tissue for histological analysis, by which blood and other fluids are first washed out by PBS and then replaced by PFA, a potential edema is cleared out and not assessable anymore.

The synchrony of the transient MPTP-induced alteration of MRE parameters and neuronal cell density in our mouse model of dopamine depletion highlights the pivotal role of neurogenesis for brain elasticity. It is remarkable that Nestin/GFP-positive precursor cells do not alter MRE constants until their differentiation into NeuN-positive neurons. Hence, their mechanical properties must differ from those of neurons. It has already been demonstrated that neuronal cells are stiffer than glial cells [41], and that the elasticity of reactive glial cells depends on intermediate filament cell content [8]. In future experiments, neurons and neural precursor cells should be studied *in vitro*, applying MRE to analyse their individual mechanical properties and using histological and biomolecular techniques to correlate these mechanical characteristics with their cellular and molecular features. Interestingly, MRE seems to be more sensitive to the process of cell differentiation than histological cell counts and provides additional information about the dissemination of neuronal turnover. Albeit most enhanced in the hippocampus, we observed a marked increase of G′, G″ and abs(G*) throughout the whole brain in a transverse image slice. This apparently diffuse pattern of transiently increased neurogenesis in the hippocampus due to MPTP treatment is a surprising result and motivates further investigations of the correlation between histology and viscoelastic parameters in different regions of the brain. With the current state of the art, MRE can provide consistent quantitative values on a global scale, i.e. considering spatially averaged constants. This permits the detection of diffuse pathology as has been demonstrated for NPH, AD and MS [11]–[14]. However, fast sampling methods and optimized reconstruction routines are being developed, which expand MRE towards a high resolution imaging modality [7], [51], [52].

When comparing human MRE with small animal studies, caution has to be taken: The higher dynamic range of MRE in the mouse (900 Hz in our study) compared to ∼50 Hz in humans may cause a shift in MRE sensitivity towards the loss properties of the tissue. Whether the complex shear modulus of brain tissue at low vibration frequencies would be sensitive to detecting the changes reported in this paper remains to be determined.

In the near future, viscoelastic constants of the brain may provide the missing link between morphometric imaging parameters and neuronal health on the cellular network level. Our results provide the first indication of the involvement of newly generated neurons into the viscoelastic matrix of the brain, corroborating the hypothesis that the neuronal network effectively contributes to the mechanical scaffold of the brain and therewith encourage further studies on humans for the clinical assessment of neural plasticity and neurodegeneration by MRE.

## Supporting Information

Figure S1 **Variation of MRE parameters due to MPTP in the whole brain.** MPTP induced a transient increase of brain elasticity and viscosity (a, b and c) at 6 dpi, while the phase angle φ (d) remained unchanged (mean±SEM). *p<0.05, **p<0.01
(TIF)
