# Supplementary figures and images for: Enhanced Adult Neurogenesis Increases Brain Stiffness: In Vivo Magnetic Resonance Elastography in a Mouse Model of Dopamine Depletion
Source: PLoS One. 2014 Mar 25;9(3):e92582. doi: 10.1371/journal.pone.0092582 (PMC3965445; doi:10.1371/journal.pone.0092582)

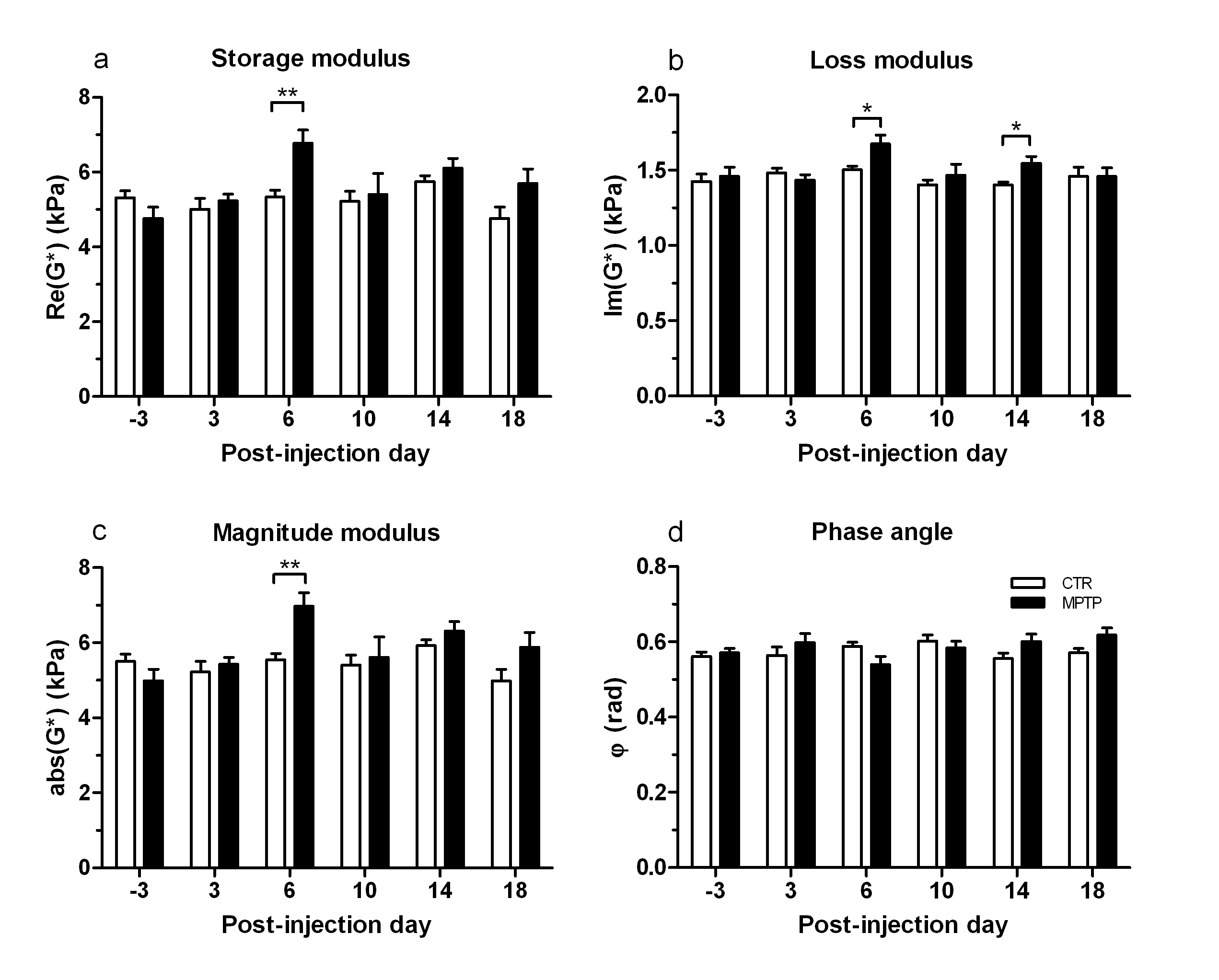

Supplement: Figure S1 — Variation of MRE parameters due to MPTP in the whole brain. MPTP induced a transient increase of brain elasticity and viscosity (a, b and c) at 6 dpi, while the phase angle φ (d) remained unchanged (mean±SEM). *p<0.05, **p<0.01 (TIF) [file pone.0092582.s001.tif]
